# A cyanobacterial photorespiratory bypass model to enhance photosynthesis by rerouting photorespiratory pathway in C_3_ plants

**DOI:** 10.1038/s41598-020-77894-2

**Published:** 2020-11-30

**Authors:** Ghazal Khurshid, Anum Zeb Abbassi, Muhammad Farhan Khalid, Mahnoor Naseer Gondal, Tatheer Alam Naqvi, Mohammad Maroof Shah, Safee Ullah Chaudhary, Raza Ahmad

**Affiliations:** 1 Department of Biotechnology, COMSATS University Islamabad, Abbottabad Campus, Abbottabad, Pakistan; 2 Biomedical Informatics Research Laboratory, Department of Biology, School of Science and Engineering, Lahore University of Management Sciences, Lahore, Pakistan

**Keywords:** Computational biology and bioinformatics, Systems biology

## Abstract

Plants employ photosynthesis to produce sugars for supporting their growth. During photosynthesis, an enzyme Ribulose 1,5 bisphosphate carboxylase/oxygenase (Rubisco) combines its substrate Ribulose 1,5 bisphosphate (RuBP) with CO_2_ to produce phosphoglycerate (PGA). Alongside, Rubisco also takes up O_2_ and produce 2-phosphoglycolate (2-PG), a toxic compound broken down into PGA through photorespiration. Photorespiration is not only a resource-demanding process but also results in CO_2_ loss which affects photosynthetic efficiency in C_3_ plants. Here, we propose to circumvent photorespiration by adopting the cyanobacterial glycolate decarboxylation pathway into C_3_ plants. For that, we have integrated the cyanobacterial glycolate decarboxylation pathway into a kinetic model of C_3_ photosynthetic pathway to evaluate its impact on photosynthesis and photorespiration. Our results show that the cyanobacterial glycolate decarboxylation bypass model exhibits a 10% increase in net photosynthetic rate (*A*) in comparison with C_3_ model. Moreover, an increased supply of intercellular CO_2_ (C_i_) from the bypass resulted in a 54.8% increase in PGA while reducing photorespiratory intermediates including glycolate (− 49%) and serine (− 32%). The bypass model, at default conditions, also elucidated a decline in phosphate-based metabolites including RuBP (− 61.3%). The C_3_ model at elevated level of inorganic phosphate (Pi), exhibited a significant change in RuBP (+ 355%) and PGA (− 98%) which is attributable to the low availability of C_i_. Whereas, at elevated Pi, the bypass model exhibited an increase of 73.1% and 33.9% in PGA and RuBP, respectively. Therefore, we deduce a synergistic effect of elevation in CO_2_ and Pi pool on photosynthesis. We also evaluated the integrative action of CO_2_, Pi, and Rubisco carboxylation activity (V_cmax_) on *A* and observed that their simultaneous increase raised *A* by 26%, in the bypass model. Taken together, the study potentiates engineering of cyanobacterial decarboxylation pathway in C_3_ plants to bypass photorespiration thereby increasing the overall efficiency of photosynthesis.

## Introduction

Plants employ photosynthesis to synthesize sugars to support their growth and survival^1^. Photosynthesis consists of light and dark reactions, which takes place in different compartments of plants^2^. Light reactions produce energy in the form of adenosine triphosphate (ATP) and nicotinamide dinucleotide phosphate reduced hydrogen (NADPH) by oxidation of water molecules in presence of light^3^. While, dark reactions converts CO_2_ into complex sugars by consuming the ATP and NADPH that has been produced by light reactions^2^. The dark reactions (also called Calvin cycle or C_3_ cycle) start with the Ribulose 1,5 bisphosphate carboxylase/oxygenase (Rubisco) enzyme, which catalyzes its substrate ribulose-1,5 bisphosphate (RuBP) by taking up CO_2_ as well as O_2_^2^. The carboxylation reaction yields two molecules of 3-phosphoglycerate (PGA, a 3 carbon compound)^4^. PGA is then utilized in the Calvin cycle for production of sugars, amino acids as well as for regeneration of RuBP^5^. Oxygenation of RuBP produces one molecule each of 2-phosphoglycolate (PG, 2 carbon compound) and PGA^6,7^. PG is a toxic compound which inhibits the activities of Calvin cycle enzymes such as Rubisco and triose phosphate isomerase^8^ and needs to be metabolized. Plants have evolved photorespiration as a mechanism to metabolize PG into PGA by a series of enzymatic reactions that take place in the peroxisomes, mitochondria and chloroplasts^5,9,10^. During photorespiration, hydrogen peroxide (H_2_O_2_) and ammonia (NH_3_) are also produced along with the loss of one molecule of fixed CO_2_ from mitochondria^10,11^.

The loss of fixed carbon molecules along with re-assimilation of NH_3_ and detoxification of H_2_O_2_ renders photorespiration a high-energy demand process^5^. Furthermore, with an increase in temperature, Rubisco’s specificity for O_2_ increases in comparison with that of CO_2_ which enhances oxygenation reaction thus adding to the cost of photorespiration^4,12^. Though photorespiration is crucial for metabolizing PG, it increases the cost of carbon fixation in photosynthesis by up to 50%^4,13^. These losses are more prevalent in C_3_ plants which lack the CO_2_ concentrating mechanism (CCM) to increase the supply of CO_2_ in the vicinity of Rubisco to suppress photorespiration^14^. This makes photorespiration an important target for modification in the C_3_ plants, to avoid carbon loss and conserve energy towards improvement of photosynthesis.

To achieve this goal, various attempts have been made to either downregulate the genes involved in photorespiratory pathway or decrease the oxygenation reaction of Rubisco^5,11^. Initial efforts in this regard aimed to identify genes that code photorespiratory enzymes such as phosphoglycolate phosphatase in *Arabidopsis thaliana*^15,16^. The resultant photorespiratory mutants, however, exhibited stunted growth, chlorosis and poor performance under ambient conditions due to the accumulation of photorespiratory pathway intermediates^15,16^. Chemical inhibition of glycolate oxidase (GO) activity in soybean resulted in a significant reduction of starch levels which suggested that metabolism of photorespiratory intermediates is essential to recycle carbon into Calvin cycle^17^. Efforts were also made to reduce oxygenation reaction by modifying enzymatic properties of Rubisco as well as engineering foreign Rubisco in plants^18,19^. Cyanobacterial Rubisco with associated chaperons was engineered in tobacco plants and the transgenic lines exhibited successful assembly of cyanobacterial Rubisco within plant chloroplast^20^. Later on, successful assembly of functional cyanobacterial Rubisco without associated proteins was also reported in tobacco^21^. In both studies, transgenic plants showed autotrophic growth, albeit at elevated CO_2_, owing to the very nature of cyanobacterial Rubisco^20,21^. However, these findings necessitate the introduction of CO_2_ concentrating mechanism (CCM) along with cyanobacterial Rubisco, to concentrate CO_2_ around Rubisco for improving photosynthesis. The limited success in abolishment of photorespiration by mutating photorespiratory pathway enzymes or Rubisco oxygenation reaction indicated that photorespiration is inevitable in C_3_ plants. This led to employment of PG metabolism rerouting strategies in order to minimize photorespiration losses^11^. The first report of photorespiratory bypass involved introduction of complete *E. coli* glycerate pathway into the chloroplast of *Arabidopsis*^22^. This bypass catabolized the glycolate (GCA, immediate product of PG) into glyoxylate (GOA), tartronic semi-aldehyde (TSA) and glycerate (GCEA)^22^. The resultant transgenic plants exhibited enhanced photosynthesis due to liberation of CO_2_ by the bypass in chloroplast. Later on, Maier et al*.* (2012) also reported the catabolism of GCA in chloroplast by using plant glycolate oxidase (GO), malate synthase (MS) and bacterial catalase (CAT)^23^. The release of CO_2_ in chloroplast enhanced the rate of carboxylation which led to improvement in photosynthetic rates^23^. Recently, South et al*.* (2019) also evaluated effectiveness of photorespiratory bypasses in field gown tobacco by engineering Kebeish et al*.*’s (2007), Maier et al*.*’s (2012) bypasses along with a modified Maier et al*.* bypass^22–24^. In the case of modified Maier et al.’s bypass, South et al*. *(2019) swapped GO with algal glycolate dehydrogenase^24^ to catabolize GCA. Additionally, glycolate flux was maximized towards the bypass by blocking glycolate-glycerate transporter through RNA interference (RNAi)^24^. The transgenic plants containing algal glycolate dehydrogenase exhibited enhanced photosynthetic rates, which was further increased by modulation of glycolate–glycerate transporter with RNAi^24^. Interestingly, Cyanobacteria also possesses three photorespiratory pathways i.e. plant like, *E. coli* like glycerate and unique glycolate decarboxylation pathway, which employ glycolate dehydrogenase (GDH) to catabolize GCA^25,26^. The glycolate decarboxylation pathway comprises of glycolate dehydrogenase (GDH), hydroxyacid dehydrogenase (HDH), oxalate decarboxylase (ODC), and formate dehydrogenase (FDH) for complete decarboxylation of GCA^25^. GDH catabolizes GCA into glyoxylate which is then catalyzed by HDH into oxalate, ODC catabolizes oxalate into formate and releases one molecules of CO_2_. Eventually, Formate is catalyzed by FDH and releases a second molecule of CO_2_^25^. Note that just like the modified Maier et al.*’s* bypass, cyanobacterial glycolate decarboxylation pathway also yields two molecule of CO_2_ as a result of GCA catabolism^24,25^. Transformation of individual genes of cyanobacterial glycerate and glycolate decarboxylation pathways in chloroplast of potato and *Arabidopsis* catabolized GCA and exhibited promising results^8,27^.

Alongside these efforts, synthetic biology approaches have become invaluable in investigating dynamical behavior of metabolic networks towards improving photosynthesis^28^. Several such kinetic models have been developed to evaluate the impact of environmental conditions on photosynthesis, distribution of plant resources to improve photosynthesis, and explore different conditions which can influence photosynthetic processes to improve plant productivity^29–33^. Xin et al*.* (2015) also developed mathematical model for Kebeish et al.’s and Maier et al.’s bypass which not only validated the experimental results but also further elucidated the potential of bypasses under different conditions^22,23,33^. Mathematical modelling of cyanobacterial glycolate decarboxylation pathway can, therefore, assist in systematic evaluation of its impact on photosynthetic processes. Such a model can provide invaluable assistance in development of a comprehensive strategy for genetic engineering of cyanobacterial decarboxylation pathway in C_3_ plants.

In this work, we report a novel kinetic model of cyanobacterial photorespiratory bypass by integrating cyanobacterial glycolate decarboxylation and C_3_ photosynthetic pathways^25,34^ and evaluate its synergistic effect on photosynthesis and photorespiration. Our results showed that the proposed cyanobacterial photorespiratory bypass successfully diverts the photorespiratory flux into the chloroplast by catabolizing GCA which resulted in production of two molecules of CO_2_. Increased availability of intercellular CO_2_ (C_i_) resulted in an increased rate of carboxylation besides reducing the level of photorespiratory pathway intermediates and phosphate based metabolites in Calvin cycle. Furthermore, an elevation of inorganic phosphate (Pi) pool augmented the level of phosphate based metabolites. Lastly, an increase in intercellular CO_2_ (C_i_) was observed to significantly enhance the net photosynthetic rate (*A*).

Taken together, our findings suggest that integration of cyanobacterial photorespiratory bypass can significantly enhance the overall rate of photosynthesis in C_3_ plants. We report that an optimal distribution of Pi is critical in maintenance of energy supply to Calvin cycle for regenerating RuBP and has a synergistic effect on photosynthesis. In conclusion, this study highlights the potential of engineering cyanobacterial decarboxylation pathway into C_3_ plants to enhance photosynthetic rates leading to better crop yields.

## Results

### Integration of the cyanobacterial decarboxylation bypass into C_3_ photosynthetic pathway catabolizes glycolate (GCA) and produces CO_2_ in chloroplast

**Figure 1:**
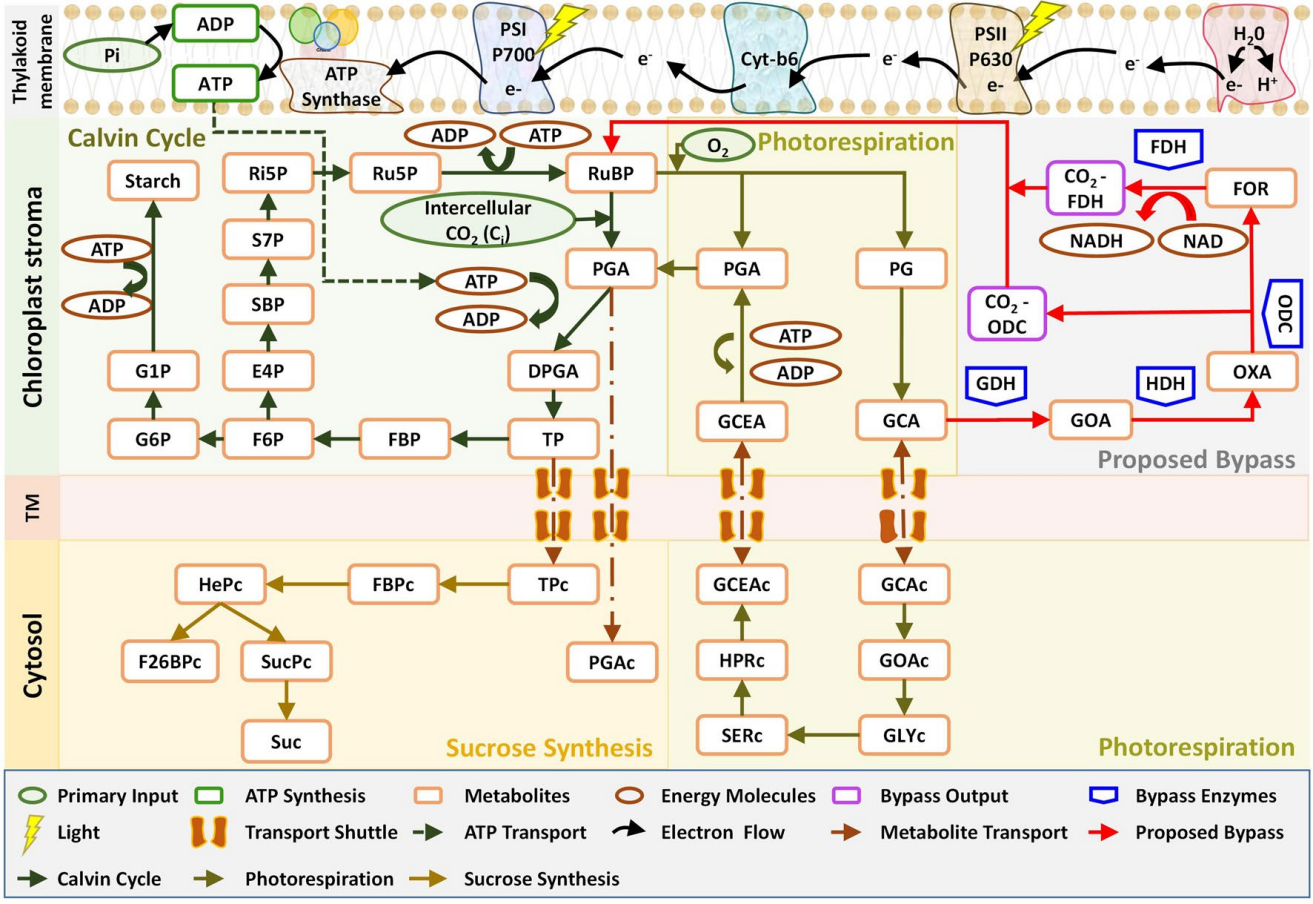
Schematic overview of cyanobacterial decarboxylation pathway’s integration into C_3_ photosynthetic pathway. The integrated pathway includes: ATP synthesis through light reaction in thylakoid membrane, RuBP carboxylation and synthesis of intermediates responsible for regeneration of RuBP, and starch synthesis through Calvin cycle in chloroplast stroma, Photorespiratory pathway which involves oxygenation of RuBP and production of respective pathway intermediates in chloroplast and cytosol, cyanobacterial decarboxylation pathway with 4 enzymes involved in GCA catabolism and generate 2 molecules of CO_2_ in chloroplast_,_ and sucrose synthesis pathway in cytosol. The compartment labelled is TM, transport membrane.

**Figure 2:**
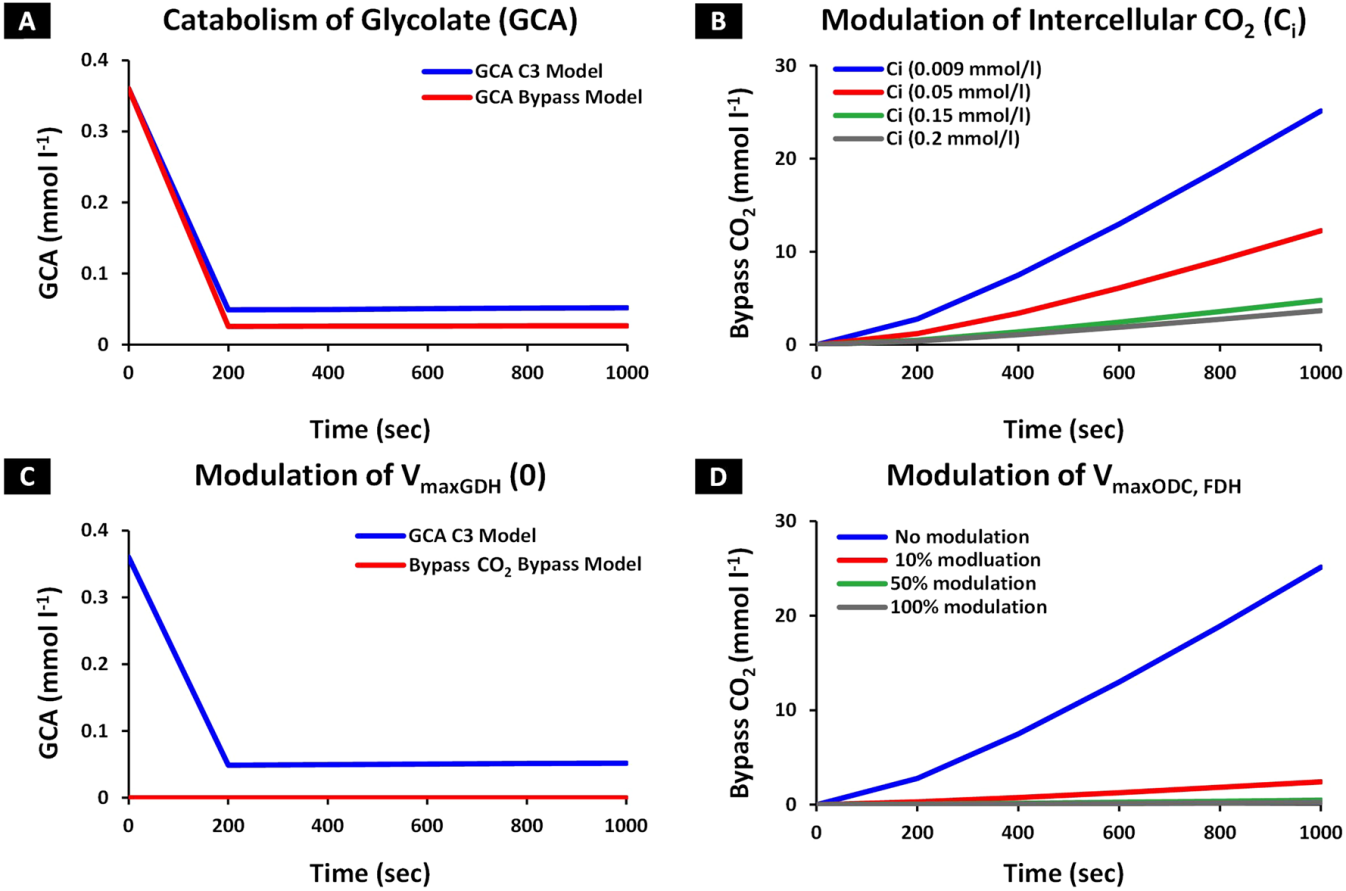
Model sensitivity analysis of the cyanobacterial glycolate decarboxylation bypass integrated into the C_3_ model. The cyanobacterial glycolate decarboxylation pathway was integrated into the C_3_ model by using the kinetic parameters of enzymes involved in the bypass to catabolize glycolate (GCA) and liberate CO_2_. (**A**,**B**) Variation in concentration of GCA and bypass CO_2_ at default and perturbed conditions of C_3_ model, over time. The cyanobacterial glycolate decarboxylation bypass was integrated into the C_3_ model to yield CO_2_ by catabolism of GCA. Variation in CO_2_ production due to catabolism of GCA at increased concentration of intercellular CO_2_ (C_i_) was also determined. In the bypass model, C_i_ concentration was varied from 0.009 mmol l^−1^ to 0.05 mmol l^−1^, 0.15 mmol l^−1^ and 0.2 mmol l^−1^ while maintaining levels of O_2_ to 0.2646 mmol l^−1^. (**A**) GCA concentration in the bypass model in comparison with reported in C_3_ model, and (**B**) CO_2_ production in the bypass model at 0.009 mmol l^−1^, 0.05 mmol l^−1^, 0.15 mmol l^−1^ and 0.2 mmol l^−1^ of C_i_. (**C**,**D**) Model sensitivity to variation in enzyme capacity (V_max_) of GDH, ODC and FDH over time. V_maxGDH_ was set at ‘0’ to shut down the bypass. V_maxODC_ and V_maxFDH_ were decreased by 10%, 50% and 100% of their respective steady state enzyme capacities (0.03 mmol l^−1^ s^−1^ and 0.015 mmol l^−1^ s^−1^). (**C**) GCA attained the steady state concentration reported in the C_3_ model and no CO_2_ production was observed in the bypass model, and (**D**) CO_2_ produced with no perturbation of steady state V_maxODC_ and V_maxFDH_, 10%, 50% and 100% decrease of V_maxODC_ and V_maxFDH_.

We integrated the cyanobacterial glycolate decarboxylation bypass^25^ into a literature-based C_3_ model^34^ that comprised of Calvin cycle, photorespiratory and sucrose pathways, towards catabolizing GCA (Fig. 1). For that, kinetic parameters of 4 enzymes including glycolate dehydrogenase (GDH), hydroxyacid dehydrogenase (HDH), oxalate decarboxylase (ODC) and formate dehydrogenase (FDH) were obtained from the literature^35–40^. Next, these parameters were tuned until the cyanobacterial photorespiratory bypass model (termed onward as ‘*bypass model*’) attained steady state. At steady state, the enzyme maximum capacity (V_max_) for GDH, HDH, ODC and FDH were 0.12 mmol l^−1^ s^−1^, 0.06 mmol l^−1^ s^−1^, 0.03 mmol l^−1^ s^−1^ and 0.015 mmol l^−1^ s^−1^, respectively. The bypass model catabolized GCA in the chloroplast and released two molecules of CO_2_ for onward uptake by Rubisco for carboxylation. GCA concentration was observed to decrease to 0.027 mmol l^−1^ in the bypass model against 0.0518 mmol l^−1^ of the C_3_ model (Fig. 2A) and 25.13 mmol l^−1^ of CO_2_ were produced by the bypass model (Fig. 2B). GCA production was regulated dynamically by the availability of substrates i.e. RUBP and O_2_. Moreover, no condition was set on oxygenation reaction in the bypass model. CO_2_ production from the bypass depends upon GCA availability to the bypass enzymes which produces two molecules of CO_2_ per GCA. Next, to validate the integration of decarboxylation bypass in the C_3_ model, we varied the concentration of intercellular CO_2_ (C_i_ = 0.009 mmol l^−1^) while maintaining level of O_2_. CO_2_ production from the bypass model decreased in comparison with the original concentration of 25.13 mmol l^−1^ which was observed using default conditions. Specifically, CO_2_ fell by 51%, 81% and 85.4% as C_i_ levels increased to 0.05 mmol l^−1^, 0.15 mmol l^−1^, and 0.2 mmol l^−1^, respectively (Fig. 2B). The decrease in the production of CO_2_ by the bypass corresponds to the low availability of GCA (catabolized by GDH) coupled with low rate of photorespiration due to an increase in concentration of C_i_. This result is in line with the published studies which report a reduced photorespiration rate followed by the decline in the production of photorespiratory pathway intermediates due to an increased concentration of C_i_^24,41,42^. Next, we evaluated model sensitivity to variability in V_max_ of GDH, ODC, and FDH enzymes while maintaining default conditions of C_3_ model. At V_maxGDH_ = 0, bypass shutdown was observed with no CO_2_ production while GCA attained the steady state concentration reported in the C_3_ model (Fig. 2C). Modulation of V_maxODC_ and V_maxFDH_ level to 10%, 50% and 100%, while maintaining V_maxGDH_ at 0.12 mmol l^−1^ s^−1^, resulted in a corresponding decrease of CO_2_ production by 90.4%, 98.1% and 99% in the bypass (Fig. 2D). These results showed that the bypass model successfully catabolized GCA and exhibited sensitivity to the model parameters, enzyme capacities of bypass enzymes, and concentration of C_i._

### Photorespiratory bypass enhances the rate of carboxylation by diverting the photorespiratory flux to the chloroplast

**Figure 3:**
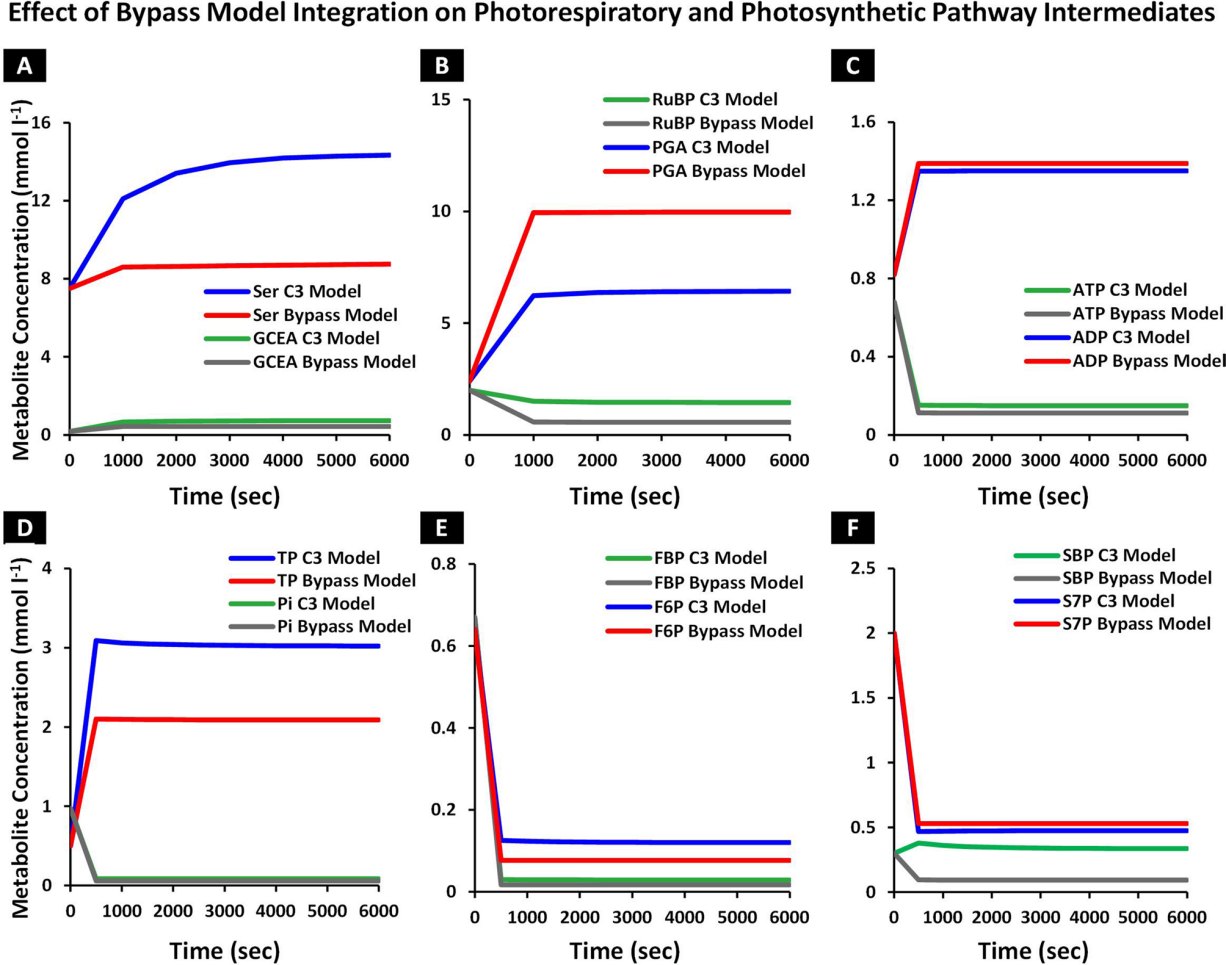
Effect of photorespiratory bypass integration into C_3_ model, on concentration of metabolic intermediates, over time. The model was run to steady state while maintaining default conditions including initial metabolite concentrations of C_3_ model and results were compared with C_3_ model. (**A**–**F**) Effect of bypass integration on the concentration of metabolites in photorespiratory pathway and Calvin cycle, over time. The model was run to steady state and concentration of serine (SER), glycerate (GCEA), phosphoglycerate (PGA), Ribulose 1,5 bisphosphate (RuBP), adenosine triphosphate (ATP), adenosine diphosphate (ADP), triose phosphate (TP), inorganic phosphate (Pi), fructose 1,6 bisphosphate (FBP), fructose 6 phosphate (F6P), sedoheptulose 1,7 bisphosphate (SBP) and sedoheptulose 7, phosphate (S7P) were determined and compared with C_3_ model. (**A**) SER and GCEA concentration in the bypass model in comparison with the C_3_ model, over time, (**B**) PGA and RuBP concentration in the bypass model against C_3_ model, over time, (**C**) ATP and ADP concentration in the bypass model in comparison with C_3_ model, over time, (**D**) Pi and TP concentration in the bypass model against C_3_ model, over time, (**E**) FBP and F6P concentration in the bypass model as compared to C_3_ model, over time, and (**F**) SBP and S7P concentration in the bypass model against C_3_ model, over time.

Following the integration of cyanobacterial photorespiratory bypass into C_3_ model, we set out to evaluate its impact on photorespiratory and Calvin cycle intermediates along with rate of carboxylation. For that, the initial metabolite concentrations set in C_3_ model, were used and the model was run over time to steady state. Next, metabolite concentrations at steady state were compared with those reported in the C_3_ model. Serine (SER), a determinant of photorespiratory flux downstream of chloroplast^22^, was observed to decrease to 8.75 mmol l^−1^ in the bypass model in comparison with 14.33 mmol l^−1^ of the C_3_ model (Fig. 3A). Alongside, a reduction in photorespiratory flux was observed in the bypass model as glycerate (GCEA) concentration dropped from 0.72 to 0.43 mmol l^−1^ (Fig. 3A). Phosphoglycerate (PGA), a product of carboxylation reaction in the Calvin cycle, was observed to increase to 9.96 mmol l^−1^ in the bypass model in comparison with 6.43 mmol l^−1^ of the C_3_ model (Fig. 3B). A concomitant reduction in the concentration of substrate (RuBP) for carboxylation reaction, was observed as its concentration dropped to 0.56 mmol l^−1^ from 1.45 mmol l^−1^ (Fig. 3B). Adenosine triphosphate (ATP), which is involved in energy consuming reactions in the Calvin cycle, decreased to 0.11 mmol l^−1^ from 0.14 mmol l^−1^ while adenosine diphosphate (ADP) increased to 1.38 mmol l^−1^ from 1.35 mmol l^−1^ of the C_3_ model (Fig. 3C). Inorganic phosphate (Pi), a major contributor in the reactions of the phosphate based metabolites in chloroplast, was observed to decrease to 0.057 mmol l^−1^ from 0.084 mmol l^−1^ (Fig. 3D). Reduction in the concentration of triose phosphate (TP), a precursor of starch and sucrose synthesis pathway, was also observed to drop to a level of 2.08 mmol l^−1^ in comparison with 3.02 mmol l^−1^ (Fig. 3D). Fructose 1,6 bisphosphate (FBP) involved in the starch synthesis, was observed to decrease to 0.016 mmol l^−1^ from 0.028 mmol l^−1^ while fructose 6, phosphate (F6P) concentration level was dropped to 0.076 mmol l^−1^ from 0.119 mmol l^−1^ (Fig. 3E). Sedoheptulose 1,7 bisphosphate (SBP), which is involved in the RuBP regeneration, decreased to 0.09 mmol l^−1^ from 0.33 mmol l^−1^ while sedoheptulose 7 phosphate (S7P) concentration level increased to 0.52 mmol l^−1^ from 0.47 mmol l^−1^ of the C_3_ model (Fig. 3F). Hence, the integration of the photorespiratory bypass diverted the normal C_3_ photorespiratory pathway flux to chloroplast and utilized the GCA to produce CO_2_ in the vicinity of Rubisco. This also caused a decline in the photorespiratory pathway intermediates concentration and a concomitant increase in the rate of carboxylation.

### Increase in inorganic phosphate pool improves the rate of carboxylation and phosphate based metabolites in the Calvin cycle

**Figure 4:**
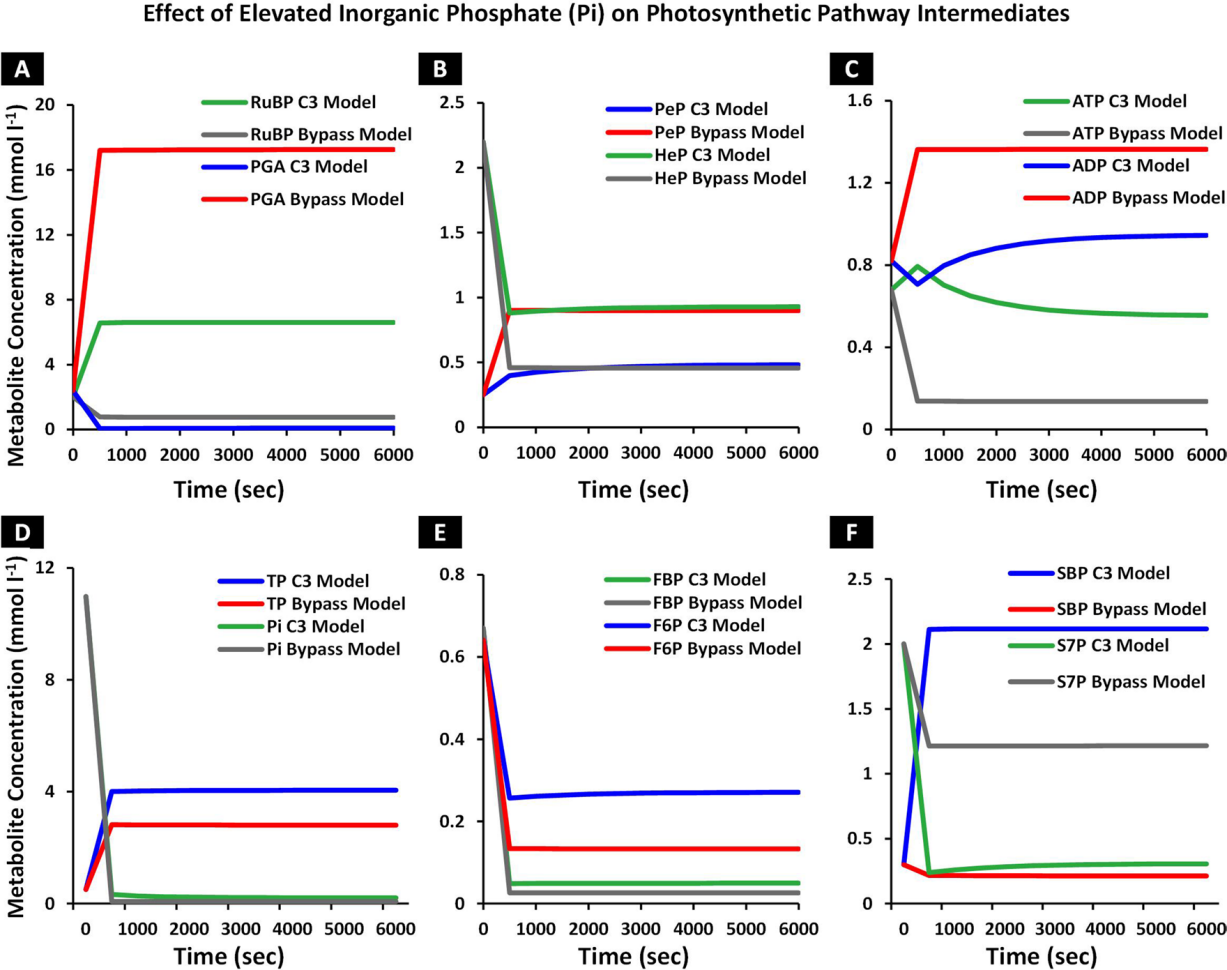
Effect of inorganic phosphate (Pi) on the concentration of phosphate based metabolic intermediates in Calvin cycle, over time. The total pool of Pi was increased to 70% in the C_3_ and bypass model. The model was run to steady state and metabolic intermediates concentration were determined in both the models. (**A**–**F**) Influence of Pi on the carboxylation rate along with starch and sucrose synthesis. The model was run over time at elevated level of Pi and concentration of phosphoglycerate (PGA), Ribulose 1,5 bisphosphate (RuBP), pentose phosphate (PeP), hexose phosphate (HeP), adenosine triphosphate (ATP), adenosine diphosphate (ADP), triose phosphate (TP), inorganic phosphate (Pi), fructose 1,6 bisphosphate (FBP), fructose 6 phosphate (F6P), sedoheptulose 1,7 bisphosphate (SBP) and sedoheptulose 7, phosphate (S7P) were analyzed. (**A**) PGA and RuBP concentration in the bypass model in comparison with C_3_ model, at elevated level of Pi, over time, (**B**) Pep and HeP concentration in the bypass model against C_3_ model at elevated level of Pi, over time, (**C**) ATP and ADP concentration in the bypass model in comparison with C_3_ model at elevated level of Pi, over time, (**D**) Pi and TP concentration in the bypass model against C_3_ model at elevated level of Pi, over time, (**E**) FBP and F6P concentration in the bypass model as compared to C_3_ model at elevated level of Pi, over time, and (**F**) SBP and S7P concentration in the bypass model in comparison with C_3_ model at elevated level of Pi, over time.

To elucidate the decline in concentration of C_3_-phosphate group intermediates in the Calvin cycle, we evaluated the impact of inorganic phosphate (Pi) pool on the metabolic intermediates. Laisk et al*.* (1986) simulated oscillations in photosynthesis by varying CO_2_ concentration and light, and reported that Pi limits photosynthesis under non-limiting conditions of these two factors^43^. Furthermore, simultaneous elevation of CO_2_ and Pi has also been reported previously in a free air CO_2_ enrichment (FACE) study which demonstrated an enhancement in the total plant biomass and phosphate (P) content in chick pea and field pea plants^44^. Therefore, to evaluate the coordinated action of enhanced supply of C_i_ by the bypass with elevated Pi, we increased the total Pi concentration (15 mmol l^−1^) by 70%^45^ in both C_3_ and bypass models and simulated each model to steady state. PGA, the immediate product of carboxylation reaction, increased to 17.25 mmol l^−1^ in the bypass model while for the C_3_ model, its concentration dropped to 0.089 mmol l^−1^ (Fig. 4A). Alongside, RuBP concentration was observed to increase to 0.75 mmol l^−1^ and 6.6 mmol l^−1^ for the two models (Fig. 4A). Pentose phosphate (PeP), pentose sugar molecule complex involved in the regeneration of RuBP, was observed to increase to 0.89 mmol l^−1^ in the bypass model while in the C_3_ model its concentration dropped to 0.48 mmol l^−1^ (Fig. 4B). Hexose phosphate (HeP) involved in the starch synthesis was observed to increase in both bypass and C_3_ models to 0.45 mmol l^−1^ and 0.92 mmol l^−1^, respectively (Fig. 4B). ATP concentration increased to 0.136 mmol l^−1^ and 0.55 mmol l^−1^ whereas ADP level dropped to 1.36 mmol l^−1^ and 0.94 mmol l^−1^ in the bypass and C_3_ models, respectively (Fig. 4C). This increase in ATP indicated that Pi regulates energy supply by photophosphorylation of ADP during photosynthesis. TP was observed to increase to 2.8 mmol l^−1^ and 4.04 mmol l^−1^ in the bypass and C_3_ models, respectively (Fig. 4D). With increasing total Pi concentration pool and its subsequent incorporation into phosphate metabolites, the Pi concentration was also observed to increase to 0.074 mmol l^−1^ in the bypass model and 0.2 mmol l^−1^ in the C_3_ model (Fig. 4D). An increasing trend was observed in FBP and F6P, FBP and F6P concentration was observed to increase to 0.026 mmol l^−1^ and 0.13 mmol l^−1^ in the bypass model while 0.049 mmol l^−1^ and 0.27 mmol l^−1^ in the C_3_ model, respectively (Fig. 4E). SBP concentration was observed to increase to 0.21 mmol l^−1^ and 2.11 mmol l^−1^ in the bypass and C_3_ models, respectively (Fig. 4F). The overall increase in the concentration of phosphate based metabolites indicates that Pi content augments the production of these metabolites during carbon metabolism. At elevated Pi, S7P concentration increased to 1.21 mmol l^−1^ in the bypass model, however it decreased to 0.3 mmol l^−1^ from 0.47 mmol l^−1^ (Pi = 15 mmol l^−1^) in the C_3_ model (Fig. 4F). A previous FACE study that also evaluated augmented phosphate (P) supply, reported an increase in total root, shoot biomass and phosphate contents, which is indicative of a synergistic action of CO_2_ and Pi on plant metabolome^44^. Furthermore, in a non-FACE study, elevation of P along with elevated CO_2_ resulted in an enhanced photosynthetic rate and plant biomass accumulation^46^. In line with these reported studies, results from our bypass model indicate that increasing the total pool of Pi together with an increased availability of C_i_ by the cyanobacterial glycolate decarboxylation pathway improves the rate of carboxylation. Additionally, an increased rate of carboxylation utilized excessive phosphate based metabolites, thus increasing the consumption of Pi which concomitantly affects phosphate based metabolites pool.

### Augmented supply of CO_2_ by cyanobacterial photorespiratory bypass enhances the net photosynthetic rate (*A*)

**Figure 5:**
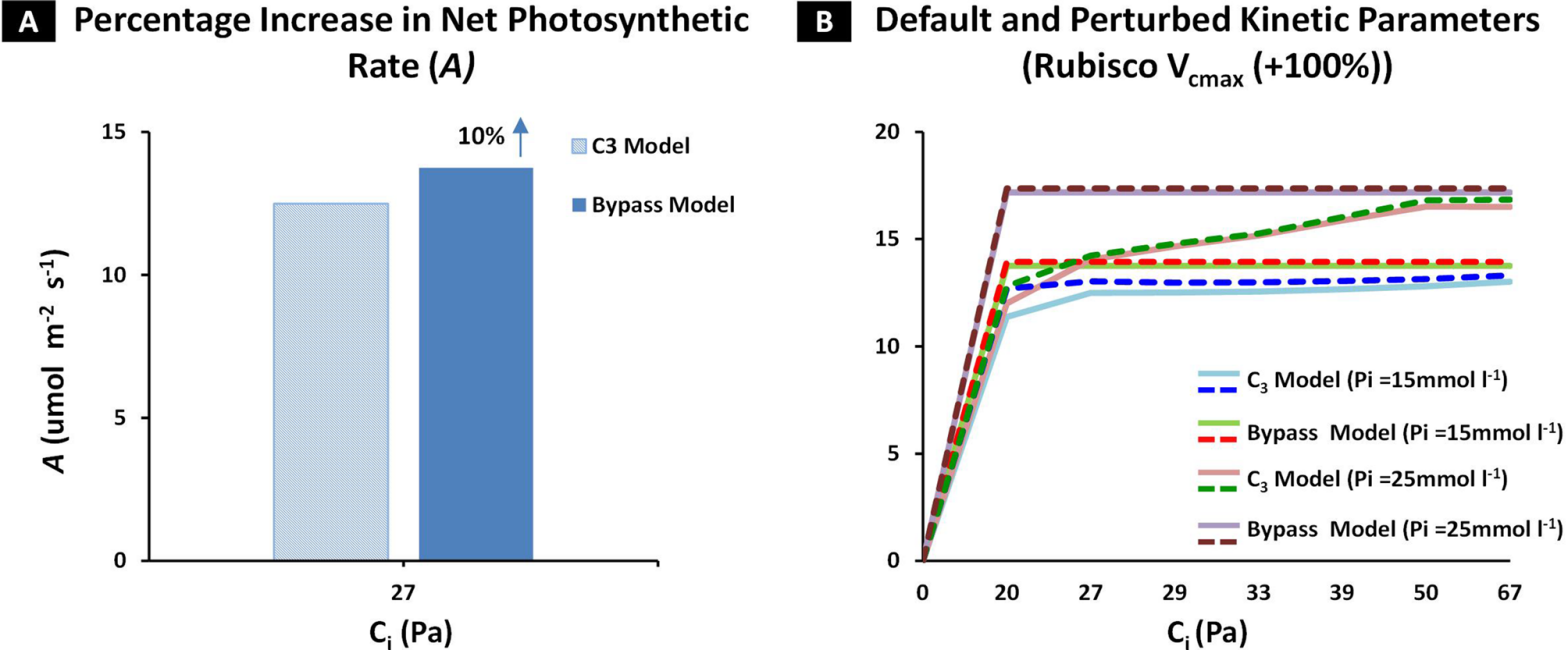
Effect of photorespiratory bypass integration on net photosynthetic rate (*A*) at different levels of intercellular CO_2_ (C_i_), Inorganic Phosphate (Pi) and Rubisco V_cmax_. The net photosynthetic rate (*A*) was determined in C_3_ and bypass model under default and perturbed conditions. C_i_ was varied from 20 to 67 Pa, Pi from 15 mmol l^−1^ to 25 mmol l^−1^ and Rubisco V_cmax_ increased to + 100% from its model default value, in both the models. *A* was computed at the steady state for each value of C_i_ for respective condition. (**A**,**B**) Effect of integrating cyanobacterial decarboxylation bypass on *A*. The model was run to steady state while maintaining the default conditions of C_3_ model and observing the change in *A* at 27 Pa Ci. Next, *A* was determined at different levels of C_i_ (20–67 Pa) for normal and elevated level of Pi (15 mmol l^−1^ and 25 mmol l^−1^) and Rubisco V_cmax_ (+ 100%). (**A**) Percentage increase in *A* reported in the bypass model in comparison with C_3_ model at 27 Pa Ci, and (**B**) *A* was determined in the bypass and C_3_ model at each C_i_ for normal and elevated levels of Pi and Rubisco V_cmax._

Having observed that an increased supply of intercellular CO_2_ (C_i_) from the bypass and elevated level of Pi (25 mmol l^−1^) resulted in an enhanced rate of carboxylation in the vicinity of Rubisco, we set out to determine the resultant effect of CO_2_, Pi and carboxylation rate on the net photosynthetic rate (*A*). For that, we used the default conditions set in the C_3_ model and measured the enhancement in *A* after the integration of photorespiratory bypass into the C_3_ pathway. A 10% increase was observed in *A* for the bypass model as compared to the C_3_ model (*A*, in the C_3_ model: 12.49 µmol m^−2^ s^−1^ and in the bypass model: 13.74 µmol m^−2^ s^−1^) (Fig. 5A). Next, to evaluate the effect of increasing levels of C_i_ on *A*, we varied the value of C_i_ from the model default value of 27 Pa to 20 Pa, 29 Pa, 33 Pa, 39 Pa, 50 Pa and 67 Pa, which reflected the estimated level of atmospheric CO_2_ (C_a_) in the years 1780, 2019, 2025, 2050, 2075 and 2100, respectively. The model was then run to steady state for each level of C_i_ and a photosynthetic CO_2_ response was obtained. In the bypass model, *A* was observed to increase rapidly to steady state value of 13.74 µmol m^−2^ s^−1^ at 20 Pa (Fig. 5B), while for the C_3_ model, it varied between 11.37 µmol m^−2^ s^−1^ at 20 Pa to a maximum of 13.01 µmol m^−2^ s^−1^ at 67 Pa (Fig. 5B). Next, we evaluated the impact of elevated levels of Pi (25 mmol l^−1^) on *A* for each value of C_i_ (20–67 Pa). The bypass model, again exhibited a rapid increase in *A* to 17.18 µmol m^−2^ s^−1^ at 20 Pa (Fig. 5B) in comparison with the C_3_ model in which case *A* increased from 12.00 µmol m^−2^ s^−1^ at 20 Pa and a maximum of 16.50 µmol m^−2^ s^−1^ at 67 Pa (Fig. 5B). Next, to determine the impact of carboxylation rate on *A*, we increased the V_cmax_ of Rubisco up to 100% from an initial 2.91 mmol l^−1^ s^−1^ in C_3_ model to 5.82 mmol l^−1^ s^−1^, at each C_i_ level (20–67 Pa). The bypass model exhibited an increase in *A* to 13.93 µmol m^−2^ s^−1^ at 20 Pa (Fig. 5B) in comparison with the C_3_ model in which case *A* was equal to 12.69 µmol m^−2^ s^−1^ and 13.30 µmol m^−2^ s^−1^ at 20 Pa and 67 Pa, respectively (Fig. 5B). Having observed an increase in *A* at elevated levels of Rubisco V_cmax_ (+ 100%) and Pi (25 mmol l^−1^) in tandem, we set out to evaluate the simultaneous effect of perturbations in Pi and Rubisco V_cmax_ on *A*. For that, the model was run to steady state for each C_i_ (20–67 Pa), together with elevated levels of Pi (25 mmol l^−1^) and Rubisco V_cmax_ (+ 100%). The bypass model exhibited an increase of 17.36 µmol m^−2^ s^−1^ at 20 Pa (Fig. 5B), while in the C_3_ model, *A* increased from 12.80 µmol m^−2^ s^−1^ at 20 Pa to 16.84 µmol m^−2^ s^−1^ at 67 Pa (Fig. 5B). Taken together, our results indicate that higher quantities of CO_2_ produced by the bypass enhanced *A* which was further amplified by increasing the level of Pi and Rubisco carboxylation velocity (V_cmax_).

## Discussion

Photorespiratory bypasses engineered into C_3_ plants have resulted in enhancement of photosynthetic rates and plant productivity^11,24,47^. Cyanobacterial glycolate decarboxylation pathway, which employs single gene coded glycolate dehydrogenase (GDH) to catabolize GCA^25,26,48^ has become an attractive target for bypassing photorespiration in C_3_ plants. Engineering of cyanobacterial GDH in C_3_ plants has also resulted in enhanced biomass production^8,27^. Therefore, a systematic evaluation of cyanobacterial decarboxylation pathway using systems biology approach can help to further elucidate its potential in improvement of photosynthesis. In this work, we have investigated the impact of engineering cyanobacterial glycolate decarboxylation pathway on photosynthesis and photorespiration in C_3_ plants through mathematical model.

Towards this goal, we have integrated cyanobacterial decarboxylation pathway into literature based model of C_3_ photosynthetic pathway and developed a kinetic model of cyanobacterial photorespiratory bypass (Fig. 1). Our model exhibited an enhanced rate of carboxylation due to an increased level of intercellular CO_2_ (C_i_) and a concomitant decline in level of photorespiratory pathway intermediates (Figs. 2A,B, 3A,B). Despite the enhanced rate of carboxylation, a decline in concentration of phosphate based metabolites within the Calvin cycle was observed (Fig. 3C–F). We further identified inorganic phosphate (Pi) pool as a key limiting factor of Calvin cycle and reported the coordinated action of elevated CO_2_ and Pi pool in enhancement of carboxylation rate and phosphate based metabolites (Fig. 4). Lastly, we also demonstrated enhancement in net photosynthetic rate *A* at elevated levels of CO_2_, Pi and Rubisco maximum carboxylation capacity (V_cmax_) (Fig. 5).

Cyanobacterial glycolate decarboxylation pathway first reported by Eisenhut et al*.* (2008) in a mutagenic study, completely detoxifies GCA utilizing GDH, and yields two molecules of CO_2_ in chloroplast^25,48^. Specifically, Kebeish et al.*’s* bypass yields one molecule of CO_2_ and glycerate (GCEA) while Maier et al.’s bypass, like cyanobacterial decarboxylation pathway, yields two molecules of CO_2_ in chloroplast^22,23^. However, Maier et al.’s bypass produces hydrogen peroxide (H_2_O_2_) as a side product during conversion of GCA into glyoxylate (GOA)^23^. H_2_O_2_ belongs to the family of reactive oxygen species (ROS) and is produced in chloroplast through electron transport chain and in peroxisomes during photorespiration^49^. In plants, under stress conditions, stomatal closure results in an enhanced rate of oxygenation which leads to overproduction of H_2_O_2_ in peroxisomes^50^_._ Under such conditions, plants engineered with Maier bypass may exhibit a higher rate of H_2_O_2_ production in chloroplast. The excess amount of H_2_O_2_ can inhibit plant growth as well as hinder the activity of Calvin cycle enzymes i.e. fructose 1,6 bisphosphatase (FBPase) and sedoheptulose 1,7 bisphosphatase (SBPase)^51,52^. Maier et al*.* engineered catalase (CAT) to scavenge H_2_O_2_^23^, however, CAT activity is inhibited at higher temperatures resulting in an over accumulation of H_2_O_2_ and triggering of the ROS cascade^53–55^. Note that, ROS is not produced during GCA catabolism by the cyanobacterial decarboxylation pathway.

South et al*.* (2019) evaluated photosynthetic rates in field grown tobacco by employing the photorespiratory bypasses reported by Kebeish et al*.* and Maier et al.^24^*.* Their work enhanced Maier et al.’s bypass and also introduced RNA interference (RNAi) to block glycolate-glycerate transporter to maximize GCA flux into the bypass^24^. Differently from the cyanobacterial glycolate decarboxylation bypass, Kebeish et al.’s bypass produced one molecule of CO_2_, while Maier et al.’s bypass produced H_2_O_2_, during GCA catabolism^22–25^. Here, it is important to note that both South et al.’s modified bypass and cyanobacterial glycolate decarboxylation pathway^24,25^ rely on GDH to detoxify GCA and yield two molecules of CO_2_ in chloroplast without any H_2_O_2_^24,25^. However, it would be interesting to evaluate the synergistic effect of blocking glycolate-glycerate transporter along with cyanobacterial glycolate decarboxylation pathway, on photosynthesis and metabolic intermediates.

Previous studies reported that integration of photorespiratory bypass enhances the rate of carboxylation due to higher concentration of C_i_ in the vicinity of Rubisco and reduction in levels of photorespiratory intermediates^22–24,27,56^. Our model also exhibited an enhanced rate of carboxylation which resulted in an increase in levels of phosphoglycerate (PGA) and a concomitant decrease in Ribulose 1,5 bisphosphate (RuBP) concentration (Fig. 3B). Moreover, a decline in GCA (− 49%) was observed in chloroplast, which confirms its catabolism by decarboxylation pathway to yield CO_2_ (Fig. 2A,B). In agreement with experimental studies^22–24,56^, serine (SER), a key determinant of photorespiratory pathway^57,58^ and glycerate (GCEA) was also observed to decrease due to diversion of normal photorespiratory flux into chloroplast (Fig. 3A). It is pertinent to note that in the C_3_ model, GCA flux from chloroplast to cytosol is regulated through kinetics of glycolate–glycerate transporter^32,34^. In case of the bypass model, the kinetics of transporter was maintained to allow the GCA flux from chloroplast into cytosol like normal photorespiratory pathway, which also provided an opportunity to evaluate the catabolism of GCA by the bypass enzymes dynamically.

The decline observed in levels of phosphate based metabolites, which stands in contradiction to experimental observations^22,23,56^ suggests an enhanced demand of Pi to keep the photosynthetic apparatus functional (Fig. 3C–F) as reported in the literature^59^. It is important to note that a decline in Pi acts to impair the photophosphorylation of ADP, which affects the ATP production thereby altering the level of phosphorylated sugars in the Calvin cycle and limiting the RuBP regeneration^46,60,61^. Under natural conditions, elevated level of CO_2_ and Pi deficiency coexist, which may reduce photosynthesis indicating an increased sensitivity of plants to phosphate stress^46^. Zheng et al*.* (2019) also reported that plants may respond adversely to elevated levels of CO_2_ and observe downregulation in photosynthesis due to nutrient unavailability^62^. These reports support our results and suggest that nutrient availability and carbon feedback mechanism both play an important role in positively regulating photosynthesis^46,63,64^.

The coordinated action of elevated level of Pi and CO_2_ in stimulating photosynthetic efficiency has been observed in free air CO_2_ enrichment (FACE) experiment conducted on legume plants^44^. Jin et al*.* (2012) reported that simultaneous elevation in CO_2_ and Pi resulted in improved P content and plant growth in chick pea and field pea^44^. At elevated level of Pi, our model exhibited an enhancement in phosphate based metabolites and *A* (Figs. 4, 5B) which conforms with the findings from the FACE experiment at elevated level of Pi^44^. In a non-FACE study, Singh et al. (2013) also reported enhancement in photosynthesis in cotton plants at elevated level of CO_2_ and P^46^. Interestingly, our model also exhibited an increase in sedoheptulose 7 phosphate (S7P) at both ambient and elevated level of Pi (Figs. 3F, 4F). Dephosphorylation rate of sedoheptulose 1,7 bisphosphate (SBP) via SBPase, to yield S7P, is regulated according to demand of S7P to regenerate RuBP^65^. RuBP regeneration is critical in maintaining functioning of Calvin cycle and avoid depletion of metabolites^66^. Higher levels of S7P can be attributed to an enhanced demand of RuBP regeneration for sustaining an increased rate of carboxylation. On the other hand, in the C_3_ model, an increase in RuBP content and a concomitant decline in S7P was observed at elevated Pi (Fig. 4A,F), which suggests a reduced utilization of RuBP owing to low availability of C_i_. Nonetheless, further investigation is required to elucidate the impact of elevated levels of Pi on S7P and other metabolites.

Our model exhibited an enhanced *A* after the integration of bypass which conforms to the previous studies^22–24,56^. However, our results show a 10% increase in *A* (Fig. 5A), as compared to 8% observed in Kebeish et al.’s bypass which employs *E. coli* glycerate pathway^22,33^. In case of Maier et al.*’s* bypass, the difference between experimental (4.34%) and kinetic model (− 31%) can be attributed to the limited rate of RuBP regeneration^11,23,33^. South et al*.* (2019) reported 24% and 18% increase in *A* with and without RNAi, respectively, for modified Maier et al.’s bypass^24^. While, for Kebeish et al.’s bypass, a 13% increase in A was observed without RNAi while RNAi led to loss in plant productivity^24^. Also in terms of plant productivity, no change was observed with Maier et al.’s bypass, both with and without RNAi^24^.

We also evaluated the response of *A* under different concentrations of C_i_ which corresponds to expected levels of atmospheric CO_2_ (C_a_) (Fig. 5B). In our model, the integration of bypass led to augmentation of the C_i_ pool in chloroplast which led to a rapid increase in *A* at 20 Pa (Fig. 5B). No change was observed with a further increase in C_i_ (27 Pa to 67 Pa) (Fig. 5B). The rapid rise and ensuing steady state of *A* can be explained by the FvCB model reported by Farquhar et al.^29^. The FvCB model hypothesized that the rate of photosynthesis can be limited either by Rubisco or by RuBP regeneration^29,67^. Rubsico-limited photosynthetic state exists when CO_2_ concentration is low and RuBP-regeneration limited state emerges at higher levels of CO_2_ due to an enhanced rate of carboxylation^68^. Bernacchi et al*.* (2013) also reported that *A* increases with an increase in C_i_ until it reaches a point of inflection beyond which a further increase in C_i_ may not enhance *A* due to limited RuBP regeneration^69^. Busch et al*.* (2017) reported that RuBP regeneration limited *A* at CO_2_ levels between 380 ppm (38 Pa) to 1000 ppm (100 Pa)^70^. This could be a possible explanation for the initial increase in *A* due to CO_2_ enrichment in our model which steadies beyond 20 Pa. On the other hand, in the C_3_ model, *A* gradually increased with increment in C_i_ concentration (Fig. 5B).

Rubisco V_cmax_ is an important kinetic parameter that regulates photosynthesis and determines the rate of carboxylation^64,71^. Our model hypothesizes that an elevation in Rubisco V_cmax_ alone does not bring a significant change in *A*, however, a simultaneous increase in Rubisco V_cmax_ and Pi resulted in a significant enhancement in *A* (Fig. 5B). This suggests that a higher Rubisco V_cmax_ requires more RuBP to enhance *A.* In 2003, Raines et al. reported that an increased photosynthetic capacity can be attained by simultaneously increasing carboxylation and RuBP regeneration^72^. An elevation in the Pi pool results in a higher regeneration of RuBP to support Rubisco’s substrate availability. Walker et al*.* (2014) described the correlation that exists between Rubisco V_cmax_ and plant nutritional status such as Pi, which influences photosynthetic rate^64^.

The proposed bypass model requires further evaluation of photorespired CO_2_ loss, ammonia (NH_3_) release and energy balance sheet to accurately estimate the benefits of the bypass in C_3_ plants. Investigation of model under enhanced photorespiratory conditions i.e. high temperature, drought and salt will help predict the response of plants in changing climatic conditions. Lastly, it is also pertinent to elucidate the interaction between nitrogen and elevated CO_2_ to analyze the impact of the bypass on nitrogen use efficiency, nutritional requirement and value in plants.

## Conclusion

The model developed in this study reveals the potential of cyanobacterial glycolate decarboxylation pathway to suppress photorespiration by catabolizing GCA in chloroplast and enhance photosynthesis. The study reports that an excess of CO_2_ requires an additional supply of nutrients such as Pi to maintain the enhanced rate of carboxylation. Furthermore, the study also emphasizes that expected elevation of atmospheric CO_2_ in the future will require an enhanced nutrient supply for regulating photosynthesis It would also be interesting to evaluate bypass engineered plants at varying concentrations of phosphate and the resultant impact on photosynthesis and plant biomass accumulation. Furthermore, elucidation of phosphate and nitrogen use efficiency along with transcriptomic, proteomic and metabolomic profiles of bypass engineered plants can provide in depth analysis of engineering photorespiratory bypasses^24^.

## Materials and methods

### Biological pathways and kinetic parameters for the model

To develop bypass model, the cyanobacterial glycolate decarboxylation pathway containing four enzymes i.e. glycolate dehydrogenase (GDH), hydroxyacid dehydrogenase (HDH), oxalate decarboxylase (ODC), and formate dehydrogenase (FDH)^25^ was integrated into the photosynthetic pathway^34^ (Fig. 1). The literature-based C_3_ model included Calvin cycle, photorespiratory and sucrose synthesis pathway^34^. The enzyme kinetic parameters for the cyanobacterial glycolate decarboxylation pathway were adopted from enzyme database BRENDA^73^ as well as from the literature. These kinetic parameters included enzyme commission (EC) no, maximum enzyme velocity (V_max_) and Michaelis–Menten rate constants (K_m_ and K_eq_) for each enzyme (Table 1). Enzymatic data on GDH, ODC and FDH (Table 1) were adopted from other bacterial species due to lack of data from cyanobacteria. For HDH, we used data on its homologue, aldehyde dehydrogenase (Table 1). V_max_ of bypass enzymes were tuned to balance the photorespiratory flux between bypass and normal C_3_ photorespiratory pathway, at steady state.

**Table 1:** Kinetic parameters of enzymes involved in cyanobacterial decarboxylation pathway.

Enzyme	EC #	V_max_ estimated (mmol l^−1^ s^−1^)	Michaelis–Menten (MM) constants (mmol l^−1^)	References
Glycolate dehydrogenase (GDH)	1.1.99.14	0.12	K_mGCA_ = 0.04	^35^
Hydroxyacid dehydrogenase (HDH)	1.2.1.3	0.06	K_mGOA_ = 0.043	^36^
Oxalate decarboxylase (ODC)	4.1.1.2	0.03	K_mOxalate_ = 4	^37^
Formate dehydrogenase (FDH)	1.17.19	0.015	K_mFormate_ = 15 K_mNad_ = 0.11 K_mCO2_ = 2.7 K_mNADH_ = 0.46 K_eq_ = 420	^38–40^

### Formulation of rate equations

Rate equations for enzymatic reactions of cyanobacterial glycolate decarboxylation pathway were developed using kinetic parameters and type of reactions i.e. irreversible and reversible reactions. All enzymatic reactions (Table 2) within the bypass obeyed the Michaelis–Menten (MM) enzyme kinetics^74^ while CO_2_ regulation reaction (Table 2) followed the law of mass action kinetics^75,76^. Standard equations for Michaelis–Menten (MM) irreversible and reversible reactions^74,77^ and mass action kinetics (k = 0.036 s^−1^)^78^ were used to develop the rate equations for GDH, HDH, ODC, FDH and CO_2_ regulation, respectively. CO_2_ regulation reaction was incorporated in the bypass model to stabilize the model and regulate CO_2_ flux within chloroplast. The change in rate of metabolite concentrations over time was determined by system of ordinary differential equations^79^, which involved the rate of reactions producing and consuming the metabolite^71^.

**Table 2:** Rate equations for reactions involved in cyanobacterial decarboxylation pathway.

Reaction name	Reactions	Rate equations
GCA dehydrogenase	GCA → GOA	$\frac{{{\text{V}}_{{{\text{maxGDH}}}} *{\text{ GCA}}}}{{{\text{K}}_{{{\text{mGCA}}}} + {\text{GCA}}}}$
Hydroxyacid dehydrogenase	GOA → oxalate	$\frac{{{\text{V}}_{{{\text{maxHDH}}}} *{\text{GOA}}}}{{{\text{K}}_{{{\text{mGOA}}}} + {\text{GOA}}}}$
Oxalate decarboxylase	Oxalate → CO_2_ + formate	$\frac{{{\text{V}}_{{{\text{maxODC}}}} *{\text{Oxalate}}}}{{{\text{K}}_{{{\text{mOxalate}}}} + {\text{ Oxalate}}}}$
Formate dehydrogenase	Formate + NAD ↔ CO_2_ + NADH	$$\frac{{\left( {{\text{V}}_{{{\text{maxFDH}}}} *\left( {{\text{Formate}}*{\text{NAD}} - \left( {{\text{CO}}_{{2}} *{\text{NADH}}/{\text{K}}_{{{\text{eq}}}} } \right)} \right)} \right)}}{\begin{gathered} ({\text{K}}_{{{\text{mFormate}}}} *{\text{K}}_{{{\text{mNAD}}}} *({1} + {\text{Formate}}/{\text{K}}_{{{\text{mFormate}}}} \hfill \\ + {\text{NAD}}/{\text{K}}_{{{\text{mNAD}}}} + {\text{CO}}_{{2}} /{\text{K}}_{{{\text{mCO2}}}} + {\text{NADH}}/{\text{K}}_{{{\text{mNADH}}}} \hfill \\ + ({\text{Formate}}*{\text{NAD}}/({\text{K}}_{{{\text{mFormate}}}} *{\text{K}}_{{{\text{mNAD}}}} )) \hfill \\ + ({\text{CO}}_{{2}} *{\text{NADH}}/({\text{K}}_{{{\text{mCO2}}}} *{\text{K}}_{{{\text{mNADH}}}} )))) \hfill \\ \end{gathered} }$$
CO_2_ regulation	CO_2_ →	K * CO_2_

### Development of the bypass model

BioModels database^80^ was used to obtain the C_3_ model (BIOMD0000000393)^34^ in system biology markup language (SBML) format, for onward integration with the cyanobacterial photorespiratory pathway^25^. All the conditions of the C_3_ model^34^ were maintained during model development and validation. COPASI (version 4.27, Build 217), an open source software supporting SBML format^81^, was used to develop the bypass model. Metabolite concentrations, rate equations and reactions corresponding to cyanobacterial decarboxylation pathway were specified under species, functions and reactions section, respectively. The concentration of glyoxylate (GOA), oxalate and formate was set to zero with simulation type ‘reaction’ to determine their level according to the rate equations defined as functions while the energy cofactor NAD/NADH was fixed at 1 mmol l^−1^^33^. For rate equations (Table 2), Henry Michaelis–Menten irreversible function^74^ and Law of mass action were selected from the functions list of COPASI, while a standard kinetic equation for reversible reaction^77^ was defined. Reactions (Table 2) were created for enzymes and regulation of CO_2_ flux. COPASI’s built in functions were then used to generate the complete set of ordinary differential equations.

### Net photosynthetic rate (*A*) computation

The FvCB model (*A* = V_c_ − 0.5 * V_o_ − R_d_)^29^ was used to compute *A,* where V_c_, V_o_ and R_d_ indicated the rate of carboxylation, oxygenation and mitochondrial respiration, respectively. The flux rate of Rubisco carboxylation and oxygenation along with ATP synthesis (representing photon flux density)^32^ at model steady state was used to compute *A* whereas R_d_ was set to 0.01 mmol l^−1^ s^−1^^29^. The model was then run to steady state and *A* was computed at model default value of C_i_ (0.009 mmol, equivalent to 27 Pa) to determine the change in *A* after integration of bypass. Photosynthesis CO_2_ response was generated by using varied levels of C_i_ which reflected the expected levels of atmospheric CO_2_ (C_a_). Variations in level of C_a_ were obtained from National Oceanic and Atmospheric Administration Earth System Research Laboratories (NOAA ESRL)^82^ for years between 1780 to 2100^62,83^. The C_i_ was then calculated (C_a _* 0.7)^83^ against each level of C_a_ and set to 20 Pa, 27 Pa, 29 Pa, 33 Pa, 39 Pa, 50 Pa and 67 Pa which corresponds to C_a_ in 1780, 2004, 2019, 2025, 2050, 2075 and 2100 respectively. Henry’s Law of partial pressure^84^ was used for inter conversion between CO_2_ gas and liquid phase while chloroplast volume^85^ was used to convert the units of *A* from mmol l^−1^ s^−1^ to µmol m^−2^ s^−1^.

### Model validation

The bypass model was simulated to attain steady state and its stability was checked. Default conditions of the C_3_ model were maintained while validating the bypass model and simulations were run using ‘Time course’ and ‘steady state analysis’ in COPASI. In time course, deterministic algorithm, LSODA was employed for solving the ordinary differential equations^86^. The bypass model was run for 6000 s by which it had attained steady state. Next, steady state and stability of the bypass model was determined by performing steady state analysis with resolution parameter set at 1e−08. The impact of elevated level of Pi and Rubisco V_cmax_ on the rate of carboxylation and metabolic levels was evaluated by varying Pi from 15 mmol l^−1^ (C_3_ model) to 25 mmol l^−1^ (+ 70%)^45^ and Rubisco V_cmax_ from 2.91 mmol l^−1^ s^−1^ (C_3_ model) to 5.82 mmol l^−1^ s^−1^ (+ 100%).

## Supplementary information

Supplementary Legend.

Supplementary Data 1.

Supplementary Data 2.
