# Supplementary material for: A cyanobacterial photorespiratory bypass model to enhance photosynthesis by rerouting photorespiratory pathway in C3 plants
Source: Sci Rep. 2020 Nov 30;10:20879. doi: 10.1038/s41598-020-77894-2 (PMC7705653; doi:10.1038/s41598-020-77894-2)
Supplement: Supplementary file 1 — Supplementary Legend. [file 41598_2020_77894_MOESM1_ESM.docx]

**Supplementary Information**

**A cyanobacterial photorespiratory bypass model to enhance photosynthesis by rerouting photorespiratory pathway in C_3_ plants**

Ghazal Khurshid^1,2^, Anum Zeb Abbassi^1^, Muhammad Farhan Khalid^2^, Mahnoor Naseer Gondal^2^, Tatheer Alam Naqvi^1^, Mohammad Maroof Shah^1^, Safee Ullah Chaudhary^2*^, Raza Ahmad^1*^

^1^Department of Biotechnology, COMSATS University Islamabad, Abbottabad Campus, Abbottabad, Pakistan

^2^Biomedical Informatics Research Laboratory, Department of Biology, School of Science and Engineering, Lahore University of Management Sciences, Lahore, Pakistan

**List of Additional Files**

**Supplementary Data 1 - C_3_ Model**

1. COPASI file
2. Steady state report generated in COPASI

**Supplementary Data 2 - Bypass Model**

1. COPASI file
2. Steady state report generated in COPASI

**Supplementary Data 1 - C_3_ Model - A COPASI file for C_3_ model along with its steady state report.** A C_3_ model of photosynthetic pathway containing Calvin cycle, photorespiratory and sucrose pathway was run in COPASI and its steady state report was generated. (01) Model file with .cps extension, and (02) Steady state report at model default conditions.

**Supplementary Data 2 - Bypass Model - A COPASI file for bypass model along with its steady state report.** Bypass model was developed by fusing cyanobacterial glycolate decarboxylation pathway into C_3_ model in COPASI. Model was run in COAPSI and its steady state report was generated. (01) Model file with .cps extension, and (02) Steady state report at model default conditions.
